# Separating the two polarities of the POLO contacts of an 26.1%-efficient IBC solar cell

**DOI:** 10.1038/s41598-019-57310-0

**Published:** 2020-01-20

**Authors:** C. Hollemann, F. Haase, M. Rienäcker, V. Barnscheidt, J. Krügener, N. Folchert, R. Brendel, S. Richter, S. Großer, E. Sauter, J. Hübner, M. Oestreich, R. Peibst

**Affiliations:** 1 Institute for Solar Energy Research Hamelin (ISFH), Am Ohrberg 1, 31860 Emmerthal, Germany; 2 Institute of Electronic Materials and Devices, Leibniz Universität Hannover, Schneiderberg 32, 30167 Hannover, Germany; 3 Laboratory of Nano and Quantum Engineering, Leibniz Universität Hannover, Schneiderberg 39, 30167 Hannover, Germany; 4 Institute for Solid-State Physics, Leibniz Universität Hannover, Appelstraße 2, 30167 Hannover, Germany; 5 Fraunhofer Center for Silicon Photovoltaics CSP, Walter-Hülse-Str. 1, 06120 Halle, Saale Germany

**Keywords:** Solar cells, Electronic devices

## Abstract

By applying an interdigitated back contacted solar cell concept with poly-Si on oxide passivating contacts an efficiency of 26.1% was achieved recently. In this paper the impact of the implemented initially intrinsic poly-Si region between *p*-type poly-Si and *n*-type poly-Si regions is investigated. Two recombination paths are identified: The recombination at the interface between the initially intrinsic poly-Si and the wafer as well as the recombination across the resulting *p*(*i*)*n* diode on the rear side which is aimed to be reduced by introducing an initially intrinsic region. By using test structures, it is demonstrated that the width of the initially intrinsic region ((*i*) poly-Si region) has a strong influence on the recombination current through the *p*(*i*)*n* diode and that this initially intrinsic region needs to be about 30 μm wide to sufficiently reduce the recombination across the *p*(*i*)*n* diode. Lateral and depth-resolved time of flight secondary ion mass spectrometry analysis shows that the high-temperature annealing step causes a strong lateral inter-diffusion of donor and acceptor atoms into the initially intrinsic region. This diffusion has a positive impact on the passivation quality at the c-Si/SiO_*x*_/*i* poly-Si interface and is thus essential for achieving an independently confirmed efficiency of 26.1% with 30 μm-wide initially intrinsic poly-Si regions.

## Introduction

An ongoing goal of the photovoltaic (PV) industry, which goes hand in hand with the energy system transformation towards renewable energy sources, is the reduction of the levelized costs of electricity^1^. An effective way to achieve this is to increase the energy conversion efficiency of solar cells and modules and thus the energy yield of a PV system. In recent times, passivating contacts in particular have contributed to those efficiency improvements^2,3^. The two approaches for passivating contacts that are mainly used are amorphous silicon (a-Si)/crystalline Silicon (c-Si)-HeteroJunctions (SHJ) and POLycrystalline silicon (poly-Si) on Oxide (POLO) junctions^4–12^. The POLO contacts consist of a thin interfacial oxide under a doped poly-Si layer, onto which the metal contacts are applied. They enable both, low contact resistances as well as a good passivation quality of the c-Si surface below the metal contacts^13–17^, thus enhancing the contact selectivity strongly as compared to conventional diffused junctions^18,19^.

Another important aspect to achieve highly efficient solar cells is the interdigitated arrangement of both contact types on the rear side of the cell. This Interdigitated Back Contacted (IBC) cell design^20^, in which both hole collector and electron collector contacts are placed on the back, completely eliminates shading losses from metal contacts and reduces parasitic absorption in doped poly-Si layers or transparent conductive oxides (in the case of SHJ) on the front side. The combination of the IBC solar cell design with passivating contacts has recently demonstrated efficiencies above 26%^21^.

An advantage of POLO junctions is their temperature stability, which implies compatibility with conventional mainstream high-temperature screen-print metallization^22,23^. The increased lateral conductivity, as compared to amorphous or nanocrystalline layers, enables low series resistances in the case of IBC cells with local contacts. However, strong recombination occurs if the highly defective *p*^+^ and the *n*^+^-type poly-Si regions touch directly^24^. This is because the high conductivity of the poly-Si does not impede a transport limitation. Therefore, a separation of the *n*-type POLO (*n*POLO) and *p*-type POLO (*p*POLO) contact fingers is required^25–29^.

**Figure 1:**
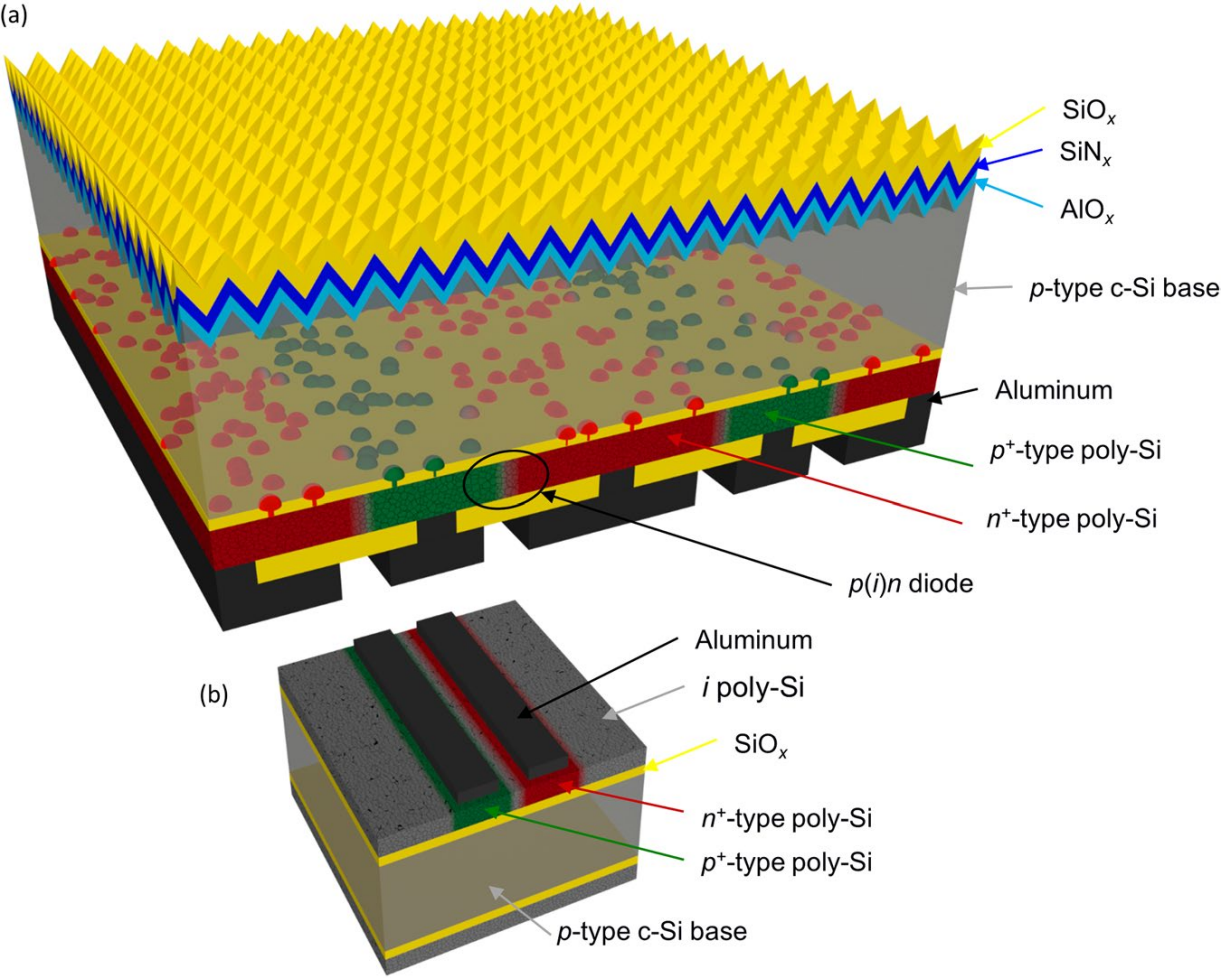
(**a**) Schematic drawing of the structure of an IBC solar cell with *n*^+^- and *p*^+^-type poly-Si contact fingers separated by an initially intrinsic poly-Si region. (**b**) Sketch of a *p*(*i*)*n* test structure.

In this work we employ a POLO junction scheme that consists of an initially full area intrinsic poly-Si (*i* poly-Si) layer that is locally doped by ion implantation. From process leanness point of view an attractive option to avoid these *pn* junctions in the poly-Si is to leave an (*i*) poly-Si region between emitter and base fingers. This is expected to result in lateral *p*(*i*)*n* poly-Si junction at the rear side of the IBC cell, which is connected in parallel to the *n*POLO/*p*-type c-Si junctions (Fig. 1a). By applying the described junction scheme we demonstrated a solar cell with an efficiency of 26.1%^11,21^.

This concept was previously tested by other groups on precursor level^30^ achieving a *V*_OC_ of 682 mV with an p*FF* of 80% and on the cell level^27^ yielding an efficiency of 18.4%. Both devices showed high ideality factors pointing to a non-ideal recombination in the space charge region. To evaluate why our cell, in contrast, does not suffer from a high ideality factor we perform a systematic study of the device physics of the resulting *p*(*i*)*n* poly-Si diodes.

We quantify the defect density in our poly-Si layers prior and after hydrogenation using time-dependent photoluminescence decay measurements in the picosecond regime.

As the purpose of the (*i)* poly-Si regions is to suppress high recombination currents across the *p(i)n* diode a large (*i*) poly-Si region is desirable. On the other hand, we observed a poor passivation quality of the c-Si absorber by the *i*POLO on full-area lifetime test structures (see below). This is due to the moderate chemical passivation of SiO_*x*_ on *p*-type Si, which further degrades when forming the nanometer-sized pinholes in the interfacial oxide^31^. This reasoning favors small (*i*) poly-Si widths. To find an optimum between these counteracting requirements, we experimentally vary the width of the (*i*) poly-Si region from nominal *d*_gap_ = 0 µm up to 380 µm.

We experimentally find that a width of the initially *i* poly-Si layer of *d*_gap_ = 30 µm together with a high annealing temperature of over 1000 °C enables a record cell efficiency of 26.1%. From measurements on full area test structures and numerical device simulations we know that the surface recombination velocity at the intrinsic poly-Si is above 2000 cm/s and would limit the device efficiency to 15%. We conclude that the nominally intrinsic poly-Si layer is no longer intrinsic after the full cell process.

We therefore investigate an inter-diffusion of dopants from the *n*-type and *p*-type doped fingers into the initially intrinsic poly-Si region by a lateral Time of Flight Secondary Ion Mass Spectrometry (ToF-SIMS) measurement. Combining all three aspects, we are, for the first time, able to present a comprehensive understanding of the working principle of high-efficient POLO IBC cells with *p*(*i*)*n* poly-Si diodes.

## Results and Discussion

Figure 1 shows the structure of the cell and the structure of the *p*(*i*)*n* diode test samples. The process flow of the test samples (see suppl. material) differs, apart from the structuring method, only by the thickness of the interfacial oxide. In order to avoid any carrier transport through the wafer the oxide in the test structures has a thickness of 100 nm. The important variation is that of the width of the nominal *i*-region *d*_gap_ of the *p*(*i*)*n* diodes, which varies between 0 and 380 µm. Furthermore, some samples are processed without the hydrogen treatment used in the cell process in order to investigate the influence of this treatment on the recombination inside poly-Si.

### Hydrogenation

The hydrogenation via a Si:H rich SiN layer and a subsequent annealing at 425 °C for 30 min, as we apply it to our cells, has the aim to passivate the recombination-active defects in the poly-Si and therefore to suppress the recombination at the *n*^+^*p*^+^ poly-Si junctions down to an acceptable level as known from monocrystalline Si^32^. If this would be successful, no further measures would be required to electrically separate the *n*^+^ and *p*^+^ doped poly-Si fingers. Furthermore, hydrogen also improves the passivation at the c-Si/SiO_*x*_/poly-Si interface^33^ and thus enhances the effective carrier lifetime in our cells.

**Figure 2:**
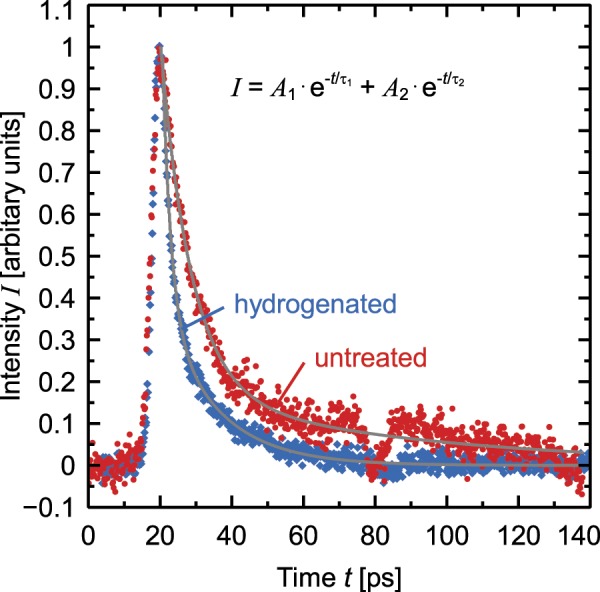
Time dependent photoluminescence signal of a poly-Si layer on a glass substrate due to an excitation with an 80 MHz Titanium-Sapphire Laser with a wavelength of 780 nm. The red circles show the signal of a non-hydrogenated sample and the blue circles show the signal of a hydrogenated sample. The grey solid lines indicate the fitted bi-exponential functions.

However, the effective carrier lifetime inside a 180 nm-thick poly-Si layer on a glass substrate is not improved due to this hydrogenation process (see Fig. 2). A bi-exponential fit yields for the untreated poly-Si a first effective lifetime component of τ_1_ = 8.2 ± 0.3 ps and a second component of τ_2_ = 65 ± 5 ps (A_1_ = 0.25 ± 0.03, A_2_ = 9.9 ± 0.8). The hydrogenated poly-Si shows a first lifetime component of τ_1_ = 2.37 ± 0.08 ps and a second component of τ_2_ = 15.0 ± 0.4 ps (A_1_ = (4 ± 1) × 10^3^, A_2_ = 1.47 ± 0.09).

The first lifetime components i.e. the lifetime inside poly-Si, determined by the time resolved photoluminescence (PL) method, changes from 8.2 to 2.4 ps due to the hydrogen treatment implying that the hydrogen does not passivate the recombination active defect states. Therefore, we conclude that the hydrogen passivation does not reduce the recombination at the *n*^+^*p*^+^ poly-Si junctions and thus the separation of the *n*^+^ and *p*^+^ doped poly-Si fingers is needed.

Beside the short-lived components a second component of 65 ps for the untreated sample can be determined from the PL-signal. As shown in Fig. 2 this long-lived component is reduced after the hydrogen treatment, which could possibly be explained by a passivation of defect-states that rather cause trapping than recombination. The influence of this passivation on the carrier mobility was first shown by Seager *et al*.^34^ who attributed it to a significant reduction of the grain boundary potential barriers.

**Figure 3:**
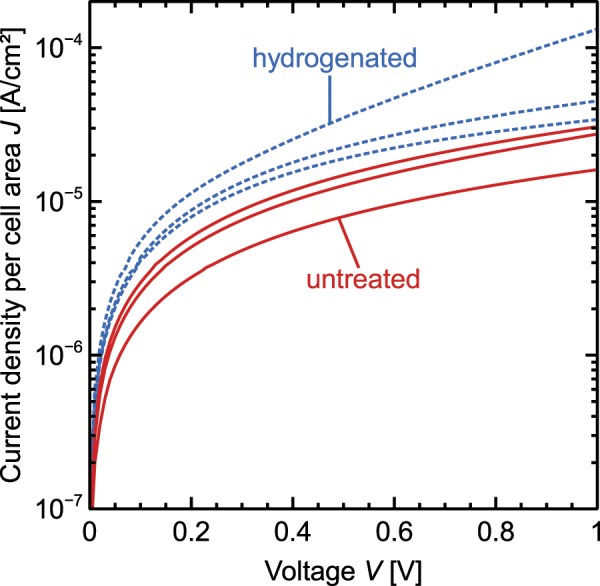
Dark-*I*-*V*-curves of *p*(*i*)*n* diode structures with a width of 50 µm of *i*-region. The dotted lines show results of hydrogenated samples and the solid lines show result of samples that have not received a hydrogen treatment.

In our *p*(*i*)*n* diode test samples a higher mobility is disadvantageous to the recombination current through the *p*(*i*)*n* diode. An increase of the dark current due to the hydrogen treatment can be seen in our data for *p*(*i*)*n* diodes having a nominal width of above 50 µm (see Fig. 3). However, as the passivation of the SiO_*x*_/c-Si interface benefits from the hydrogen treatment – the effective lifetime increases from 1.1 ms to 1.5 ms in the reference sample with *p*POLO and from 3.1 ms to 3.2 ms in the reference sample with *n*POLO — we put up with the slightly higher dark *I*-*V*-currents and yet hydrogenate our cells.

### Current voltage characteristics of *p*(*i*)*n* test structures

**Figure 4:**
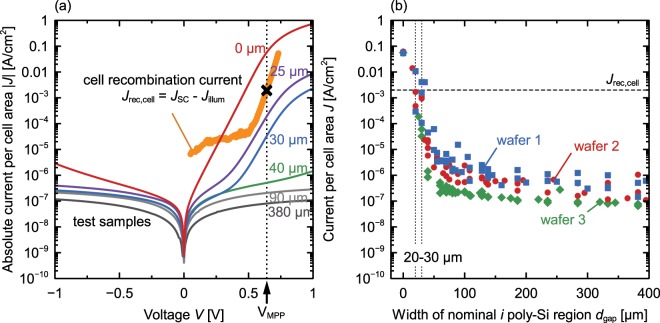
*J*-*V* analysis of the *p*(*i*)*n* test structures. (**a**) *J*-*V* curves of *p*(*i*)*n* diodes with different width of the (*i*) poly-Si region. The *MPP* and *J*_*rec*_ refer to the record solar cell with an initially 30 µm-wide intrinsic region. (**b**) Current per cell area *J* through the *p*(*i*)*n* diodes at a voltage of 0.64 V (representative for the maximum power point of our solar cells) as a function of the designated width of the intrinsic region. The different colors indicate three identical test structures on different wafers.

Figure 4a shows the results of the current voltage measurements of samples having received a hydrogen treatment, as in the cell process. To enable a comparison between the current density through the *p*(*i*)*n* diode and the total recombination current density of the solar cell, Fig. 4 gives the currents calculated per front surface area. A solar cell with a nominal 30 µm wide *i*-region, poly-Si thickness of 150 nm and a cell area of 4 cm^2^ contains a lateral *p*(*i*)*n* junction area of 2.7 × 10^−3^ cm^2^ which gives a factor of 6.7 × 10^−4^ for the current density compared to the current per vertical *p*(*i*)*n* junction area of test structures (9 × 10^−6^ cm^2^).

The poly-Si *pn* diode (zero width of intrinsic region) shows a high current density of up to *J*_0 µm_ = 60 mA/cm^2^ at a voltage of 0.64 V (*V*_MPP_ of the final solar cell). Regarding small *i*-regions of nominally up to 25 µm and 30 µm the current per cell area at *V*_MPP_ decreases to *J*_25 µm_ = 0.2 mA/cm^2^ and *J*_30 µm_ = 0.03 mA/cm^2^ at *V*_MPP_ while the diode-like behavior remains.

In the range between nominally 30 µm and 40 µm both, the absolute value of the current density and the shape of the *J*-*V* curve change drastically. The asymmetrical diode behavior changes towards a more symmetric one. The current density drops by about two orders of magnitude to a value of 5 × 10^−7^ A/cm^2^ at *V*_MPP_. For further enlargements of the nominal *i*-region width up to 380 µm the current density decreases by about one further order of magnitude. All *J*-*V* curves with a 90 µm or larger nominal *i*-region width do not show rectifying behavior.

Beside the *J*-*V* curves of the test structures, Fig. 4a also displays the maximum power point (*MPP*) of the solar cells at 0.641 V and 40.6 mA/cm^2^. The total recombination current density *J*_rec_ = *J*_SC_ − *J*_illum_ of this cell can be calculated from the difference between the short circuit current density *J*_SC_ and the current density under one sun illumination *J*_illum_ and applied bias. By comparing this with the recombination current through the nominal *i*-region, we conclude that a nominal *i*-region width of 30 µm is enough to reduce the latter to an extent that does not significantly compromise the cell performance anymore.

### Dependence of recombination current on the width of the nominal*i*-region

Figure 4b shows the current per cell area through the *p*(*i*)*n* test structures, at 0.64 V, as a function of the nominal width of the (*i*) poly-Si region. From 20 μm to 40 μm, the current density decreases steeply. A clear correlation between the recombination current and the nominal (*i*) poly-Si region width can be seen. Most of the data points for diodes with *d*_gap_ between 20 µm and 30 µm are smaller than the total recombination current density *J*_rec_ of the record cell^11^. Those widths correspond to the nominally 30 µm-wide *i*-region of the record cell.

### Passivation quality of intrinsic poly-Si

To estimate the impact of an *i*-region on the rear surface passivation quality, we measure the carrier lifetimes using the photoconductance decay method on symmetric reference samples with full area *p*^+^-type poly-Si, *n*^+^-type poly-Si and *i*-type poly-Si processed in this cell batch. Table 1 shows a very low effective lifetime of 9 µs at the MPP in the *i*POLO sample while the two samples with doped POLO junctions exhibit effective lifetimes of 1400 µs and 3300 µs respectively.

**Table 1:** Effective lifetimes measured on symmetric full area reference wafers with *i*POLO, *n*POLO and *p*POLO junctions after hydrogenation.

Reference sample	*τ*_eff_ at MPP of the cell [ms]
*i* poly-Si	0.009
*p*^+^ poly-Si	1.392
*n*^+^ poly-Si	3.289

We simulate the unit cell of our POLO-IBC solar cells to estimate the impact of such a poor lifetime on the cell performance. The simulations are performed in Quokka 2^35,36^, which employs our conductive boundary model^37^. We calculate an injection-dependent surface recombination velocity *S*_eff_ from the injection-dependent lifetime measured on the *i*POLO sample assuming a bulk Shockley-Read-Hall lifetime of 100 ms.

**Figure 5:**
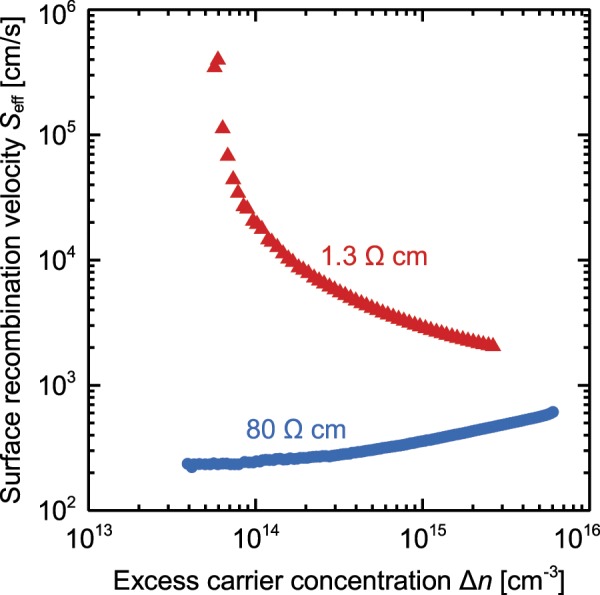
Surface recombination velocity (SRV) *S*_eff_ as a function of the excess carrier concentration and the resistivity of *p*-type silicon wafers with *i*POLO junctions.

**Figure 6:**
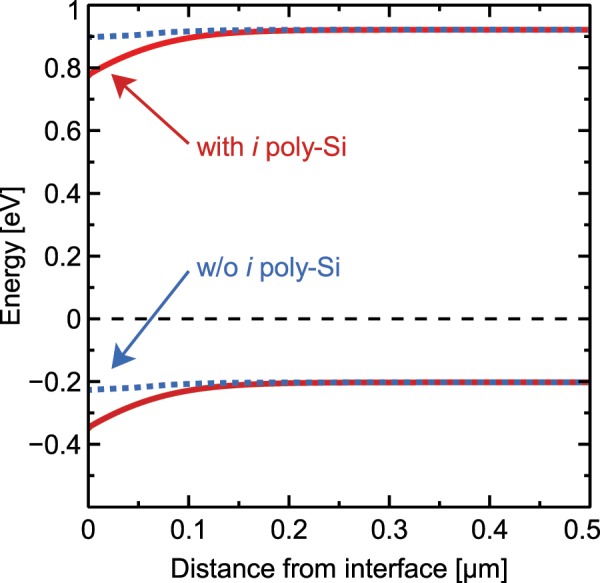
Band diagram at the interface to the silicon oxide with and without *i* poly-Si on top.

Figure 5 shows these *S*_eff_ values of the *i*POLO reference samples on 1.3 Ω cm and 80 Ω cm material. We observe a rising SRV with increasing doping level that was previously also shown for bare thermally oxidized *p*-type wafers^38^. This effect can be explained by positive fixed oxide charges *Q*_f_ that cause an inversion at the surface in lightly doped *p*-type silicon wafers which develops towards depletion with increasing doping level. This inversion condition is even enhanced, when intrinsic poly-Si is applied on top of the oxide, which leads to a stronger recombination and therefore even higher *S*_eff_ values. The corresponding band diagrams (see Fig. 6) have been calculated with the semiconductor module of Comsol Multiphysics, using the common models for numerical device simulations of solar cells^39^ and assuming a fixed charge density of 5 × 10^10^ cm^−2^ adjacent to the silicon wafer.

All the other simulation parameters for the description of our cell can be found elsewhere^40^. The simulation shows that the *i*POLO junctions with a width of d_gap_ = 30 µm, covers 13.3% of the rear side area and limits the efficiency potential to 14.9%. The discrepancy between the simulated efficiency of 14.9% and the actual measured efficiency of the cell of 26.1% shows that the *S*_eff_ value in the cell differs from that on the test structures. The simulations of the 26.1%-efficient cell^40^ indicates that the passivation quality in the intrinsic gap regions is between *S*_eff_ of 15 and 20 cm/s.

### Density of pinholes and their impact on the passivation quality of intrinsic poly-Si

Determining the areal densities of the pinholes helps to understand whether the high recombination activity at the c-Si/SiO_*x*_/*i* poly-Si interface is exclusively caused by the moderate chemical passivation of SiO_*x*_ on *p*-type Si^38^ or possibly also by the formation of pinholes^31,41–43^ in the interfacial oxide. We apply selective etching^42^ followed by an investigation with Scanning Electron Microcopy (SEM) to determine the pinhole densities. The resulting pinhole areal densities are listed in Table 2. Differences between the *p*-type, *n*-type and intrinsic POLO junctions and the etching times can be seen. The base doping however has no effect on the pinhole areal density.

**Table 2:** Etch pit areal density (*EPD*) in the interface oxide of a *p*^+^, *n*^+^ and *i*POLO junction after appropriate etching times.

Doping type	Resistivity [Ω cm]	*EPD* [cm^−2^]	*J*_0_ [fA/cm^2^]	Etching time [min]
*i* poly-Si	1.3	2.6 ± 0.1 × 10^6^	2900	4
*i* poly-Si	80	2.8 ± 0.2 × 10^6^	540	5
*n*^+^ poly-Si	80	1.0 ± 0.6 × 10^8^	6	1
*p*^+^ poly-Si	80	1.96 ± 0.09 × 10^7^	5	5

**Figure 7:**
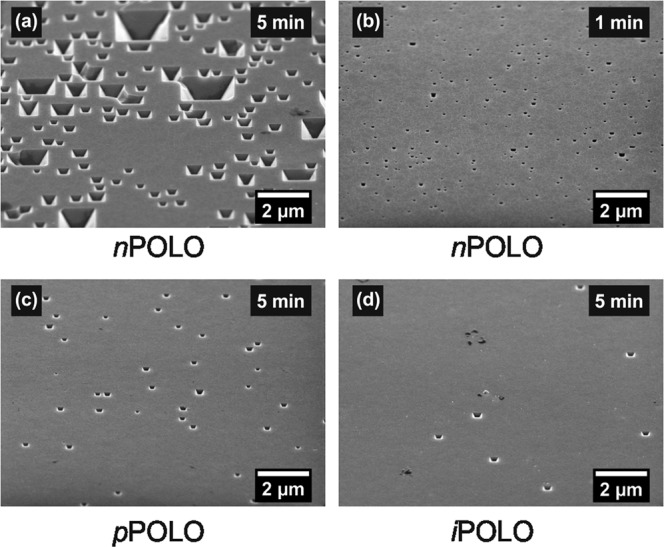
SEM images of full area reference samples on 80 Ω cm *p*-type wafers after etching with 15% TMAH at 80 °C. The samples are tilted 31° out of the vertical position. In image (**a**) after etching for 5 min the etch pit are so wide that they are growing into one another, which would falsify the areal density. Thus, we chose a shorter etching time of 1 min (**b**) for the evaluation of this sample. In image (**c**,**d**) the etching time of 5 min is appropriate for determining the pinhole areal density.

In Fig. 7 SEM images of the *n*POLO, *p*POLO and *i*POLO samples etched for 5 min and of the *n*POLO sample etched for 1 min are shown. Figure 7a of the *n*POLO sample etched for 5 min shows that the etch pits have reached a size so that several of them grew into each other. An evaluation of this images would thus underestimate the etch pit density. Therefore, a suitable etching time is chosen for each sample. To eliminate the influence of local differences in the pinhole density we investigate an area of 1000 µm^2^ on each sample. The *n*POLO contact contains the largest pinhole areal density of (1.0 ± 0.6) × 10^8^ cm^−2^ (1 min etched sample). The *p*POLO sample has a lower pinhole density of (1.96 ± 0.09) × 10^7^ cm^−2^ and the *i*POLO samples shows the lowest pinhole density of (2.8 ± 0.2) × 10^6^ cm^−2^ on an 80 Ω cm wafer and (2.6 ± 0.1) × 10^6^ cm^−2^ on a 1.3 Ω cm wafer.

**Figure 8:**
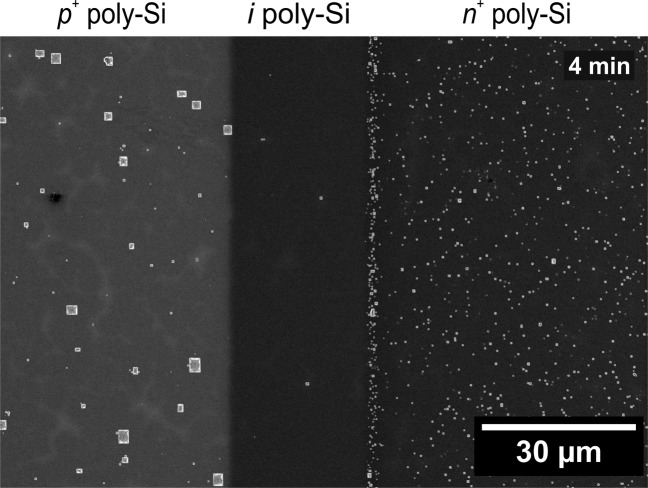
SEM images of a *p*(*i*)*n* junction on cell wafer on 1.3 Ω cm *p*-type material after etching with 15% TMAH at 80 °C for 4 min.

Figure 8 shows a SEM image of a *p*(*i*)*n* junction on a cell wafer. This image confirms that the differences between those regions in pinhole density are also present inside the cells. However, what differs here from the full area samples is the size of the pinholes. In the p-type region there are very large pinholes of up to 3 µm size which do not appear on the full area pPOLO samples. Moreover, the reason for accumulation of pinholes at the interface to the nPOLO region is also not clear.

In terms of the pinhole current transport picture, a doping of the poly-Si is essential for introducing carrier selectivity^18,44^. Intrinsic regions therefore do not show carrier selectivity and thus allow for high recombination at the interface. The *i*POLO sample shows considerably fewer pinholes than the doped POLO samples. The recombination activity at the still present pinholes will, however, be larger than for the *p*POLO and *n*POLO junctions due to the missing asymmetry of electron and hole concentrations and thus a negligible carrier selectivity.

To estimate the recombination activity in the pinholes we calculate the saturation current density as a function of the pinhole areal density using the Fischer model^41,45^. For this we assume a pinhole radius *r*_pinhole_ of 2 nm following the transmission electron microscope (TEM) investigations which was done by Tetzlaff *et al*.^46^ and Peibst *et al*.^41^. We exclusively consider here recombination in the pinholes, i.e., we assume a surface recombination velocity of zero for the passivated regions.

**Figure 9:**
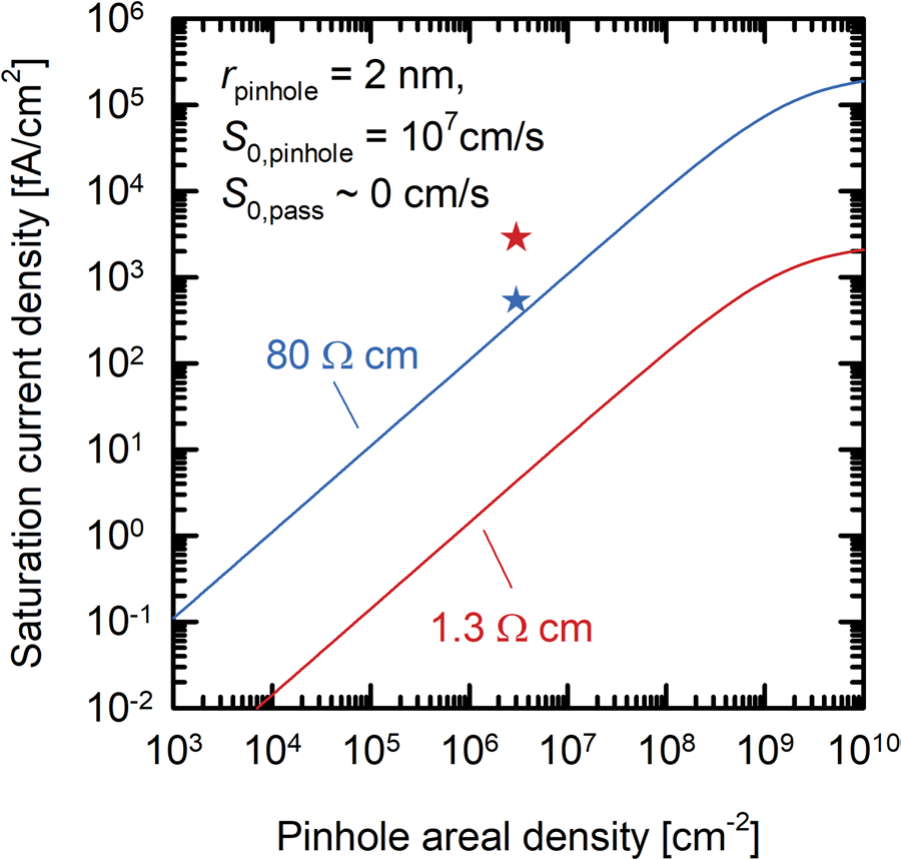
Saturation current density as a function of the pinhole areal density determined using the given parameters together with the Fischer model. The blue and red lines present the two different base materials that were used for the cells and the reference wafers. The stars indicate the saturation current densities calculated from the measured lifetimes and the respective measured pinhole areal densities.

Figure 9 shows that the simulated saturation current density decreases with the doping concentration for all densities of the pinholes. The measured saturation current density *J*_0_ of the 80 Ω cm sample (blue star) lies only slightly above the simulated value. The saturation current density of the 1.3 Ω cm sample (red star) is about three orders of magnitude larger than the simulated value. Thus, the differences between the measured and simulated values are due to the recombination at the c-Si/SiO_*x*_ interface. The interfacial recombination is thus clearly dominating in the 1.3 Ω cm sample and is at least of similar size as the recombination in the pinholes in the 80 Ω cm sample.

### Comparison of large area and small area, embedded (*i*) poly-Si regions by effective lifetime mapping

**Figure 10:**
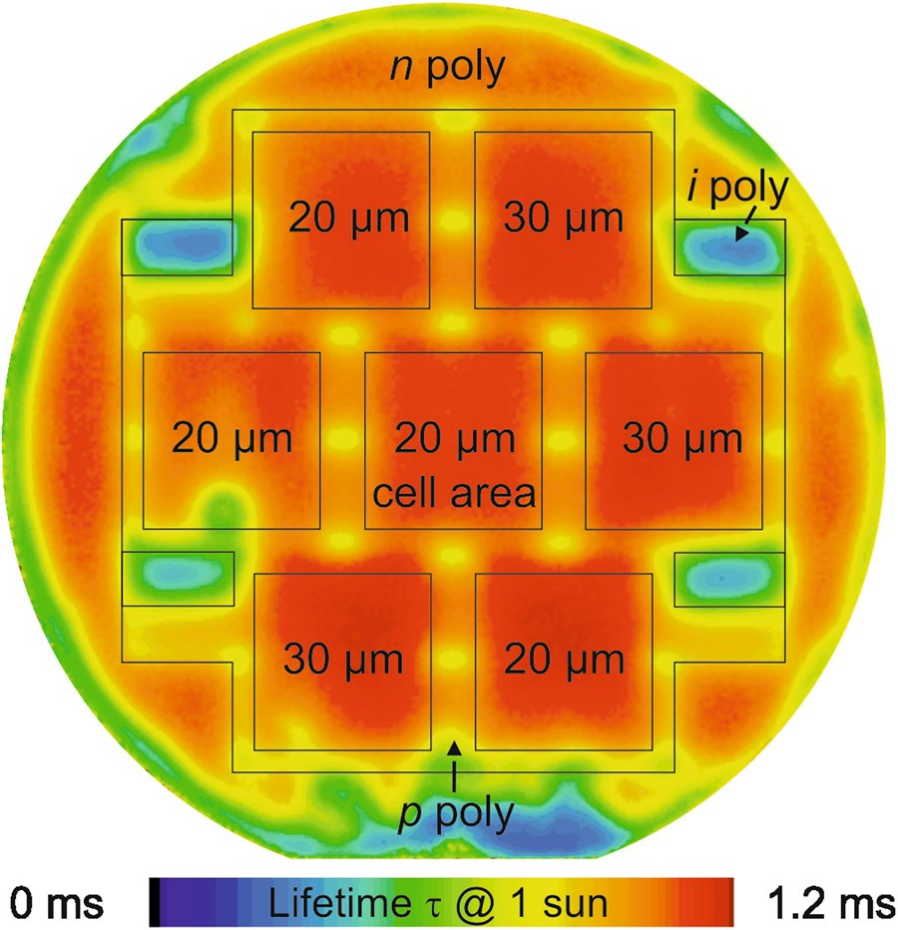
Spatially resolved carrier lifetime of a cell at 1 sun measured by the ILM method. The active cell areas of the seven solar cells are marked with squares and the value inside gives the nominal width of the *i*-region. The area between the cells is *p*^+^-type doped poly-Si. The four smaller regions contain intrinsic poly-Si.

To track down this apparent contradiction between the actually achieved efficiency and the one simulated based on the *S*_eff_ values of the full-area *i* poly-Si reference samples, we record a spatially resolved infrared lifetime mapping image of cell precursors in the state before metallization (see Fig. 10). The image shows poor lifetimes and thus high SRV in the four large *i*-regions at the corners. The cell areas containing an interdigitated *p*^+^- and *n*^+^-finger structure with either 20 µm or 30 µm nominal *i* poly-Si regions show the highest lifetimes.

### Lateral inter-diffusion of dopants in the embedded (*i*) poly-Si regions

Those high lifetimes in the cell areas indicate that the small-area (*i*) poly-Si regions, embedded between the *n*^+^ and *p*^+^ poly-Si fingers, are qualitatively different to the four large area *i*-regions. An inter-diffusion of the dopants into the initially intrinsic embedded regions during the high temperature treatment (1035 °C for 1 h) of the POLO contacts could explain this finding. The enhancement of the diffusion coefficients *D* of boron and especially phosphorous in poly-Si of about four orders of magnitude, as compared to the respective values in crystalline Si (at a temperature of 1050 °C of *D*_P_ = 1.4 × 10^−9^ cm^2^/s^47^ and *D*_B_ = 6.3 × 10^−12^ cm^2^/s^48^), supports this hypothesis. We conduct lateral and depth-dependent ToF-SIMS measurements on a solar cell precursor that is annealed at 1035 °C and has 30 µm-wide nominal *i* poly-Si regions to clarify if lateral diffusion actually occurs.

**Figure 11:**
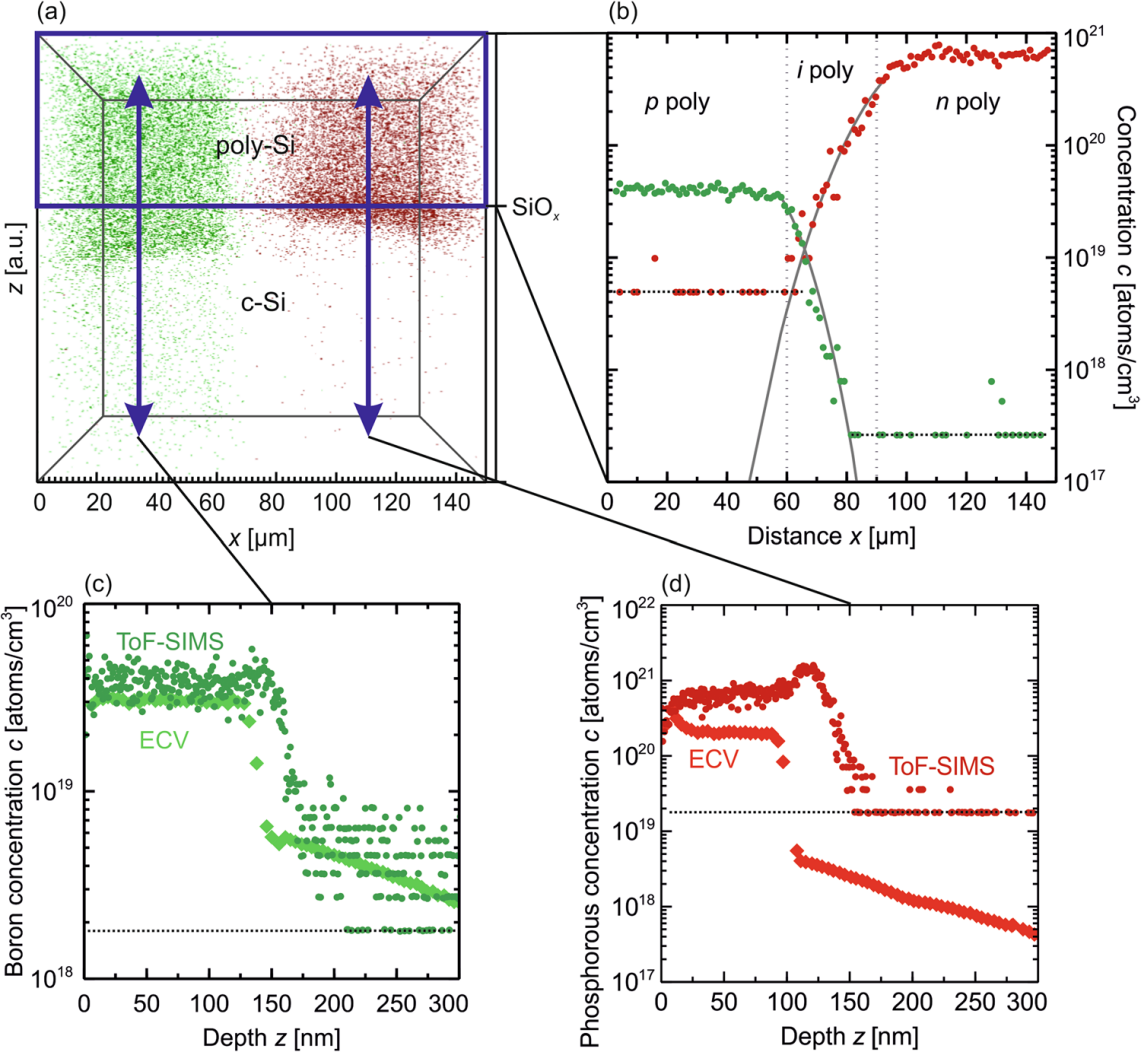
ToF-SIMS analysis of a *p*(*i*)*n* structure in a solar cell. (**a)** 3d point diagram of the spatial resolved dopant concentrations of boron and phosphorous in a volume with an area of 140 × 140 µm^2^ and a depth of 314 nm. (**b**) Summed doping concentration over volume in Fig. 10a above a depth of 100 nm. The grey solid lines show exponential fits to the data in the formally intrinsic region to quantify the inter-diffusion. (**c**,**d**) Depth profiles of the boron and phosphorous concentration determined by ToF-SIMS and ECV measurements. The difference in position in z-direction of the decrease of the dopant concentrations need to be investigated further. The resolution limits of the different TOF-SIMS measurements are marked by black dotted lines.

As shown in Fig. 11c, the vertical boron concentration and the vertical electrically active boron concentration, respectively, as measured by the Electrochemical Capacitance-Voltage (ECV) method and by a ToF-SIMS. Both techniques match well and yield a mean concentration of (4±1) × 10^19^ cm^−3^.

The mean vertical phosphorous concentration in Fig. 11d however differs by about a factor of two between the ECV (2.5× 10^20^ cm^−3^) and the vertical ToF-SIMS measurement (6× 10^20^ cm^−3^). Moreover, the phosphorous profile from the ToF-SIMS measurement shows a distinct peak at the depth of 120 nm from the surface, which coincides with the position of the interfacial oxide. This finding can have different origins. We speculate that at least part of the higher signal is due to the high oxygen content in the SiO_*x*_ layer. In the positive ionization mode, oxygen leads to an increased signal and thus to an overestimation of the dopant concentration.

The uncertainty of the P concentration is even higher than that of B^49^ because the ^31^P signal is overlaid by the signal of the ^30^Si^1^H molecule. In addition to these artifacts, an actual pile up of dopants at the SiO_*x*_ interface, which thus become electrically inactive, cannot be excluded. A 80 times higher segregation coefficient of P matches the significantly large peak as compared to B^50^.

The lateral and depth-dependent measurement was carried out over the entire volume shown in Fig. 11a with an area of 150×150 µm^2^ and a depth of about 314 nm. The summed doping concentration over the whole surface area in y-direction and up to a depth of 100 nm of this volume is shown in Fig. 11b. We limit the summation to above the depth of the phosphorous peak as the extent of the phosphorous peak that is increased by the higher oxygen content would falsify the mean doping concentration. The measurement reveals a phosphorous concentration of 5 × 10^20^ cm^−3^ in the *n*^+^-type poly-Si region and a boron concentration of 4 × 10^19^ cm^−3^ in the *p*^+^-type poly-Si regions. In the region that was initially undoped prior to the annealing step, we see an exponential decay of the dopant concentrations from the adjacent regions. The boron concentration decreases faster with the distance from the *p*^+^-type poly-Si region having a diffusion coefficient of about (8 ± 2) × 10^−11^ cm^2^/s. The phosphorous diffusing significantly further gives a fitted diffusion coefficient of (2 ± 1) × 10^−10^ cm^2^/s. The intersection of the boron and phosphorous concentration curves is located at a distance of 24 μm from the *n*^+^-type region and a distance of 4 μm from the *p*^+^-type region at a concentration of 2 × 10^19^ cm^−3^. These profiles explain the improved passivation quality of these nominally *i*-regions as compared to large and thus actual *i*-regions on the same wafer (Fig. 11).

Even a light doping of the poly-Si with phosphorus drastically improves the passivation quality of the POLO junctions of both recombination paths: pinhole recombination is reduced by the formation of a *pn* junction in the vicinity of the pinholes^18,31^ and the recombination at the Si/SiO_*x*_ interface is reduced by an introduction of a certain band bending. In the regions where the poly-Si is eventually *p*-type doped an accumulation of holes and a reduction of the electron concentration occurs at the c-Si side of interface. In the regions where the poly-Si is eventually *n*-type doped an inter-diffusion of phosphorous into the c-Si occurs, leading to an increase in electron concentrations and a reduction of the hole concentration on the c-Si side of the interface. In both regions, the effective c-Si/SiO_*x*_ interface recombination is reduced as compared to the situation in large area *i* poly-Si regions, where the c-Si side of the interface is depleted and both carrier types are present at a similar concentration. Not only the absolute amount of the c-Si/SiO_*x*_ interface recombination is reduced, but also the voltage dependence of this recombination path is altered towards lower ideality factors. We speculate that an incomplete doping of the *i*-region by lateral inter-diffusion could be the reason for the moderate ideality factors determined on cell and precursor level in refs. ^27,30^.

The sudden decrease of the recombination current density measured on the *p*(*i*)*n* diodes with increasing *i*-region width from 30 µm and 40 µm (see Fig. 4a) is consistent with this lateral-diffusion, as a doping concentration below 1 × 10^19^ cm^2^/s that would occur for a 40 µm wide *i*-region gives reason to expect a transport limitation of the recombination current due to a drastic decrease in mobility^51^.

### Correlation between the pseudo implied efficiency and the i-region width

The two opposing recombination paths, the recombination at the (*i*) poly-Si/SiO_x_/c-Si interface and the recombination through the *p*(*i*)*n* diode, give rise to the assumption that there is an optimum in *i*-region width. At larger *i*-region widths the inter-diffusion is not strong enough to increase the doping level inside the whole nominal *i*-region to a level so that recombination at the c-Si/SiO_*x*_ interface is suppressed. If the width of this region becomes to small the inter-diffusion forms a direct *p*^+^/*n*^+^ poly junction, which again facilitates strong recombination inside the poly-Si.

To find this optimal width for our cell process we process cell precursors with a wider range of *d*_gap_ from 5 to 40 µm. The results from infrared lifetime mapping images of FZ wafers we annealed at 1035 °C like our record cell are shown in Table 3. The results scatter strongly, which is presumably due to a significant variation in lifetimes that we observe across the wafers. Nevertheless, it can be said that *i*-region widths of 5 and 10 µm are leading to significantly lower effective charge carrier lifetimes, than wider *i*-regions. Thus, those widths are too small to suppress the recombination inside the poly-Si sufficiently as there will be a very high doping level across the whole nominally intrinsic region. The cells with larger *d*_gap_ values show higher pseudo implied efficiencies with a slight positive trend towards wider *i*-regions. Up to a width of *d*_gap_ = 40 µm the recombination at the c-Si/SiO_*x*_ interface does not seem to increase due the slight decrease of the doping concentration that should still be well above 1 × 10^18^ cm^−3^.

**Table 3:** Average measured implied pseudo efficiency of *p*-type POLO-IBC cells.

Wafer type	Quantity	(*i*) poly-Si width [µm]	ipη [%]
FZ	3	5	23.6
FZ	6	10	23.8
FZ	9	15	24.6
FZ	12	20	25.2
FZ	12	25	24.6
FZ	9	30	25.0
FZ	6	35	25.6
FZ	6	40	25.4
Cz	5	5	24.2
Cz	5	10	24.6
Cz	7	15	24.9
Cz	7	25	24.4
Cz	2	35	24.6
Cz	2	40	24.3

Beside the cells on FZ wafers we also process cells on CZ material with a wet-chemically grown interfacial oxide requiring a lower annealing and break up temperature of 860 °C where the inter-diffusion should be noticeably weaker. Those cells show an optimum at an *i*-region width of 15 µm. Here too, a width of 10 µm is too small to sufficiency reduce the strong recombination inside the poly-Si. Above a width of 15 µm the effective lifetimes already start to decrease again. This can be explained by an increasing recombination at the c-Si/SiO_*x*_ interface due to an insufficient doping inside the nominally *i*-region. It becomes clear, that such a low annealing temperature of 860 °C leads to a rather small process window for the optimal width.

### Cell results

**Figure 12:**
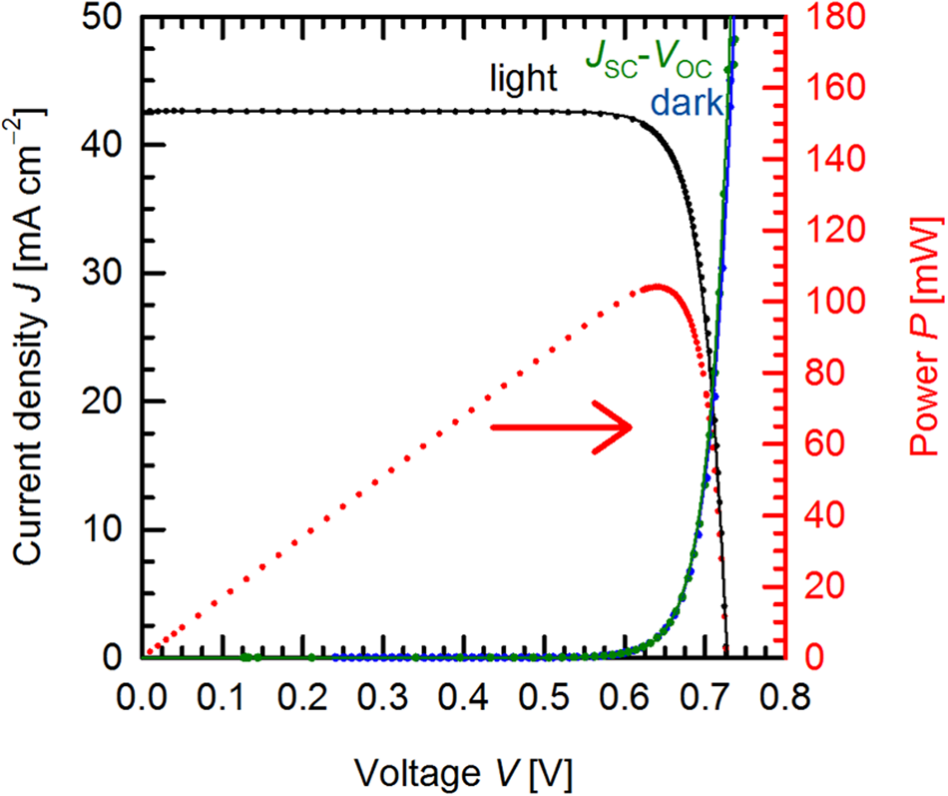
Light *J*-*V*, dark *J*-*V* and *J*_SC_-*V*_OC_-curves of the POLO-IBC cell. The dots represent the measurements and the lines the fit of the two-diode model.

The rather large inter-diffusion of the dopants which causes the selectivity to the initially *i*-region and improves the passivation quality resolves the apparent contradiction between the expectations from the Quokka simulations – based on the *S*_eff_ values for *i*POLO as determined on full area reference samples – and the independently confirmed efficiency of 26.1%^11^ for one of the cells on the wafer shown in Fig. 10. This efficiency is the second highest reported efficiency so far for Si single junction cells (in terms of team ranking), and the current world record on *p*-type Si material. Figure 12 shows the light and dark *J*-*V* and *J*_sc_-*V*_oc_ curves of the record cell, as well as the parameters extracted from the fit based on the two-diode model.

## Conclusion

We investigate the working principle of an initially intrinsic (*i*) poly-Si region between *p*^+^-type poly-Si and *n*^+^-type poly-Si regions in IBC solar cells with POLO passivating contacts. These charge carrier separating regions are a straight forward method to avoid a lateral *p*^+^*n*^+^ poly-Si junction between the base and emitter poly-Si fingers leading to high recombination currents.

We directly measure the effective lifetime in the defect-rich poly-Si by time-resolved photoluminescence. The resulting lifetime of 8.2 ps cannot be improved by the applied hydrogenation process. Therefore, a lateral *pn* poly-Si junction – i.e., the scenario without a nominal *i*-region – causes a high recombination current.

When varying nominal *i* poly-Si region widths in poly-Si *p*(*i*)*n* diode test structures we observe a strong decrease of the *p*(*i*)*n* diode recombination current density *J* with increasing *i*-region width. We find that for the oxide a nominal *i*-region width of 30 µm is enough to reduce the recombination current through this region to about two orders of magnitude below the total recombination current of the solar cell so that it is not limiting the device performance anymore.

On the other hand, we observe a very poor passivation of the crystalline Si absorber by the *i* poly-Si on full-area lifetime test structures. These high interface recombination velocities can only partially be explained by the verified formation of pinholes in the interfacial oxide. Since they are comparable to the respective values reported for *p*-type-Si/SiO_*x*_ interfaces, the majority of the interface recombination seems to be implied by the moderate chemical passivation of SiO_*x*_ on *p*-type c-Si. A numerical simulation of the cell employing these experimentally determined recombination velocities and an *i*-region width of 30 µm yields an efficiency potential of 14.9%. Comparing this to the actual efficiency of our cell of 26.1% and regarding the spatial resolved lifetime image of the cell wafer, it becomes clear that the passivation of the c-Si absorber by the (*i*) poly-Si areas embedded in the *p*(*i*)*n* diodes is much better than on full-area lifetime test structures.

Our hypothesis explaining this observation that during the high-temperature POLO-junction formation step a lateral diffusion of the *p*^+^-type and *n*^+^-type dopants into the initially intrinsic region takes place is verified by a lateral and depth resolved ToF-SIMS measurement. The measurement results confirm that an inter-diffusion generates dopant concentrations above 1 × 10^19^ cm^−3^ in an adjacent, former undoped region of up to 30 µm and thus introduces selectivity to this region, which enables high lifetimes. This in turn can explain the high energy conversion efficiency of 26.1% we achieved when applying a nominal *i* poly-Si width of 30 µm to a POLO IBC solar cell.

In addition, it remains to be investigated for which doping concentration the recombination current through the diode reaches the order of magnitude of a direct *p*^+/^*n*^+^-junction. Therefore, a cell structure with a lower boron doping followed by a masked phosphorous doping, leaving out the initially intrinsic regions, could be applied. Therefore, the doping concentration needs to be adjusted so that it remains below the doping threshold at which the recombination via the diode reaches the order of magnitude of a direct *n*^+^/*p*^+^ junction and it at the same time avoids a strong recombination at the poly-Si/SiO_*x*_/c-Si interface. This approach in turn would open up the possibility of using counter-doping^32,52^ and could at the same time ease the need for a very precise alignment.

The gained understanding will pave the way for a strongly simplified fabrication of Si solar cells with efficiencies above 26%. With this cell concept high efficiencies can be achieved while avoiding process steps like trench formation and additional dielectric passivation resulting in a much less process complexity. This could be accomplished by the combination of intrinsic poly-Si deposition with printed doping sources or structured doped glasses. However, the precise alignment that is necessary to archived the appropriate distances between *n*^+^ and *p*^+^ contacts^11,53^ still needs to be demonstrated.

All in all, this cell concept has a simple process flow that manages without TCOs and shows significant efficiency advantages over PERC.

## Methods

### Sample processing

#### Solar cells

Figure 1(a) shows the structure of an IBC solar cell with an (*i*) poly-Si region between *p*^+^-type poly-Si and *n*^+^-type poly-Si regions. First, a 2.2 nm-thin interfacial oxide layer is thermally grown onto a 100 mm-sized *p*-type FZ wafers with a resistivity of 1.3 Ω cm and a thickness of 300 µm. The oxide is subsequently capped by a 225 nm-thick low-pressure chemical vapor deposited (LPCVD) *i* a-Si layer. The local doping is generated by masked ion implantation of boron and phosphorous into the a-Si on the rear side. The photoresist implant barriers are generated by photolithography which guarantees a high accuracy and are designed in such a way, that an *i* poly-Si gap remains between the *n*^+^-type and *p*^+^-type poly-Si fingers. We would like to remark that these process steps could be eventually substitutes by industrial feasible approaches such as masked ion implantation or printing of doping sources. The finger pitches are 300 µm or 450 µm, whereas the *i* poly-Si gaps have a width of 20 µm or 30 µm. In a tube furnace, during a high-temperature step at 900 °C for 30 min the a-Si is crystallized and its surface is oxidized. A subsequent process at higher temperatures (1035 °C for 1 h) breaks up the interfacial oxide layer forming the POLO contacts. We would like to remark that the temperature budget is optimized for our rather thick thermally grown interfacial oxide. For a wet-chemically grown interfacial oxide, the temperature budget can be strongly reduced without scarifying passivation quality^54^. We hydrogenate the POLO junction by introducing hydrogen to the poly-Si/c-Si interface via a-Si:H rich SiN_*y*_ layer and a subsequent annealing at 425 °C for 30 min. Afterwards we remove the SiO_2_ layer on the front side and texturize it using a KOH-based solution. We define the contact area by laser contact opening (LCO)^11^. To passivate the front side, a 20 nm-thick atomic layer deposited (ALD) AlO_*x*_ layer is applied and capped by a plasma enhanced chemical vapor deposited (PECVD) SiN_*y*_/SiO_*z*_ layer stack. Finally, the rear side of the cell is metallized by an evaporated aluminum layer and a sputtered SiO_*z*_ layer. Here again we use a laser to pattern the SiO_*z*_ that is serving as an etching mask to separate the contacts by a wet chemical etching step^11^. Furthermore, using the same front-end process, we fabricate reference wafers with full area doped and intrinsic POLO contacts. Also, for the metallization, we are eventually targeting industrial processes such as screen-printing. Our LCO process enables the usage of non-firing through screen-print pastes, which contact the poly-Si without a degradation of its passivation quality^55^.

#### Test structures

Figure 1(b) shows the structure of the test samples which we prepare for our investigations of the *p*(*i*)*n* diodes. We start with a 180 µm thick *n*-type Cz Si wafer onto which we thermally grow a 100 nm-thick oxide to avoid any carrier transport through the wafer. Subsequently, a LPCVD-deposited intrinsic a-Si layer follows. We locally implant phosphorous and boron, masked by two masking steps using inkjet printing. Unfortunately, this method results in wavy edges, so that deviations of up to 10 µm per edge occur. The resulting *p*(*i*)*n* diodes are 6 mm long and the *p*^+^-type doped and *n*^+^-type doped regions are 1 mm wide. The width of the initially intrinsic region between the *p*^+^-type and *n*^+^-type doped areas varies nominally between *d*_gap_ = 0 µm and 380 µm. After ion implantation, the samples are annealed under oxidizing atmosphere for 30 min (900 °C plateau) followed by 1 h inert annealing (1050 °C plateau). Half of the wafers were hydrogenated via a Si:H rich SiN layer and a subsequent annealing at 425 °C for 30 min equally to the cells. Finally, we deposit Al onto the samples and separate the contacts in a wet chemical etching bath using a patterned SiO_x_ layer as an etching barrier. Apart from the *p*(*i*)*n* structures, *p*(*i*)*p* and *n*(*i*)*n* structures were processed. The *i* poly-Si gaps were varied between 0 µm and 380 µm though with a greater interval. Those structures are used to determine the resistivity of the (*i*) poly-Si.

Moreover, we have prepared *i* poly-Si samples for the time resolved photoluminescence measurement. We start with a quartz glass substrate onto which a 30 nm thick SiN is deposited followed by a 160 nm poly-Si layer. In addition, one part of the sample was subsequently hydrogenated via a-Si:H rich SiN_*y*_ layer and a subsequent annealing at 425 °C for 30 min.

### Measurements

#### Dark *J*-*V* curves of test structures

To determine the current-voltage behavior of the test diodes, we measure the dark *J*-*V* curves between −1 V to 1 V for different (*i*) poly-Si region widths by using a Süss PA 200 Probe station and a Keithley 4200 parameter analyzer.

#### Carrier lifetime inside c-Si

We measure the excess charge carrier lifetime of the *p*^+^-, *n*^+^-type and intrinsic reference wafers as well as of the realized solar cells. For this, we use the spatial resolved infrared lifetime mapping method (ILM)^56,57^. The injection dependent lifetime of the full area reference wafers is also determined by the photoconductance decay (PCD) method with a Sinton lifetime tester^58^.

#### Carrier lifetime inside poly-Si

Moreover, we determine the carrier lifetime inside a poly-Si layer on a glass substrate by means of time resolved photoluminescence spectroscopy. The sample is excited using a mode-locked Titanium-sapphire laser with 80 MHz pulse rate tuned to 780 nm wavelength. A lens is used to adjust the spot size to about 1 mm for optimal signal yield. The streak camera mechanism uses a multiplier-channel-plate (MCP) which is sensitive to the single photon level. The photoelectrons generated by the MCP are deflected by an electric field alternating with a high frequency and detected by a CCD using phosphorescent filter allowing for picosecond time resolution.

#### Light *J*-*V* characterization

The calibrated light *I-V*- and spectral response measurements on the full solar cell are performed at the Calibration and Test Center at ISFH (ISFH CalTeC). ISFH CalTeC is accredited for the calibration of solar cells according to DIN EN ISO/IEC 17025 by the German Accreditation Body (DAkkS). The fit of the *I-V* curve based on the two-diode model is performed by using the software Solar Cell Analysis (SCAN).

#### Pinhole areal density

To determine the pinhole density in the interfacial oxide in dependence of the doping level and type we use the etching method, which was first used for this purpose by Tetzlaff *et al*.^42^. Thus, we first remove the native oxide from full-area doped reference wafers using a 1% hydrofluoric acid (HF) solution. Afterwards the poly-Si and the c-Si are selectively etched in a 15% tetramethylammonium hydroxide (TMAH) solution at 80 °C. The etch rates for etching c-Si and poly-Si are more than two orders of magnitude larger than the etch rate for SiO_*x*_. Underneath the pinholes, the c-Si is exposed to the TMAH and as soon as the poly-Si is removed, etching pits emerge in the c-Si. From each reference wafer, three samples are prepared, and the etching time is varied between one and five minutes.

The size of the etch pits increases with the etching time and rapidly exceeds the size of the original pinhole. In contrast to the very small pinholes with diameters in the range of 2 nm^41^, we are able to detect the enlarged etch pits by using a scanning electron microscope (SEM). Thus, we can determine the pinhole density on the *p*, *i* and *n* poly-Si reference wafers.

#### ToF-SIMS measurement

A chemical characterization was performed by time-of-flight secondary ion mass spectrometry (TOF-SIMS) via a TOF.SIMS V (Iontof) apparatus. The lateral and depth resolved distribution of doping elements in the *p*(*i*)*n* region were analyzed at different sample positions by depth profiling mode using the sputter sources Cs^+^ (@ 1 keV) and O_2_^+^ (@ 1 keV) for negative and positive ion mode, respectively. The sputter area was chosen to 220 × 220 µm and the target area was set between 80 × 80 µm^2^ up to 150 × 150 µm^2^. For the quantification of the B^+^ and P^+^/P^−^ ion intensities, ToF-SIMS relative sensitivity factors (RSFs) regarding the Si^+^/Si^−^ intensity as reference signal are used. The primary sputter gun is Bi^+^ (@ 25 keV, bunched mode). The RSFs were previously obtained by ion implanted mono-Si standard samples. The data evaluation of the 3D ion information of each measurement at the *p*(*i*)*n* region was either integrated of the whole measured depth for the total target area or by using of regions of interest (ROI) to analyze the local depth resolved doping concentration of B and P. Additionally, lateral x-area gradients of the dopants are extracted of the ion images (integrated intensities of depth profiles below oxygen rich depth around 100 nm) to estimate the carrier concentrations in the *p*(*i*)*n* transition region.
